# Decoding allosteric landscapes: computational methodologies for enzyme modulation and drug discovery

**DOI:** 10.1039/d4cb00282b

**Published:** 2025-02-14

**Authors:** Ruidi Zhu, Chengwei Wu, Jinyin Zha, Shaoyong Lu, Jian Zhang

**Affiliations:** a Medicinal Chemistry and Bioinformatics Center, Shanghai Jiao Tong University, School of Medicine Shanghai 200025 China jian.zhang@sjtu.edu.cn; b College of Pharmacy, Ningxia Medical University Yinchuan Ningxia Hui Autonomous Region 750004 China lushaoyong@yeah.net; c State Key Laboratory of Oncogenes and Related Genes, Key Laboratory of Cell Differentiation and Apoptosis of Chinese Ministry of Education, Shanghai Jiao Tong University, School of Medicine Shanghai 200025 China

## Abstract

Allosteric regulation is a fundamental mechanism in enzyme function, enabling dynamic modulation of activity through ligand binding at sites distal to the active site. Allosteric modulators have gained significant attention due to their unique advantages, including enhanced specificity, reduced off-target effects, and the potential for synergistic interaction with orthosteric agents. However, the inherent complexity of allosteric mechanisms has posed challenges to the systematic discovery and design of allosteric modulators. This review discusses recent advancements in computational methodologies for identifying and characterizing allosteric sites in enzymes, emphasizing techniques such as molecular dynamics (MD) simulations, enhanced sampling methods, normal mode analysis (NMA), evolutionary conservation analysis, and machine learning (ML) approaches. Advanced tools like PASSer, AlloReverse, and AlphaFold have further enhanced the understanding of allosteric mechanisms and facilitated the design of selective allosteric modulators. Case studies on enzymes such as Sirtuin 6 (SIRT6) and MAPK/ERK kinase (MEK) demonstrate the practical applications of these approaches in drug discovery. By integrating computational predictions with experimental validation, this review highlights the transformative potential of computational strategies in advancing allosteric drug discovery, offering innovative opportunities to regulate enzyme activity for therapeutic benefits.

## Abstract 2

Allosteric regulation is a fundamental mechanism in enzyme function, enabling dynamic modulation of activity through ligand binding at sites distal to the active site.

## Graphical abstract

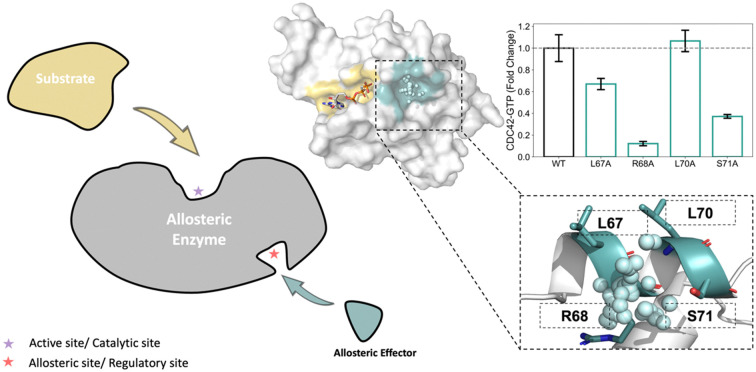

## 1. Introduction

Allosteric regulation is a pivotal mechanism through which cellular systems integrate external signals and fine-tune biological processes, enabling the dynamic modulation of metabolic pathways in response to environmental changes.^1^ Jacob and Monod first discovered that allosteric effectors attach to specific sites on proteins, distinct from the active site, thus modifying their functional properties.^2^ Such regulation is consistent with established models like cooperativity and induced fit, where the binding of an effector leads to conformational changes or adjustments in protein dynamics, often without significant structural alterations. Allosteric regulation can be classified into K-type, which affects ligand-binding affinity, and V-type, which alters the catalytic rate of enzyme. This form of regulation is critical for maintaining cellular homeostasis and coordinating complex biological functions.^1^

**Fig. 1:**
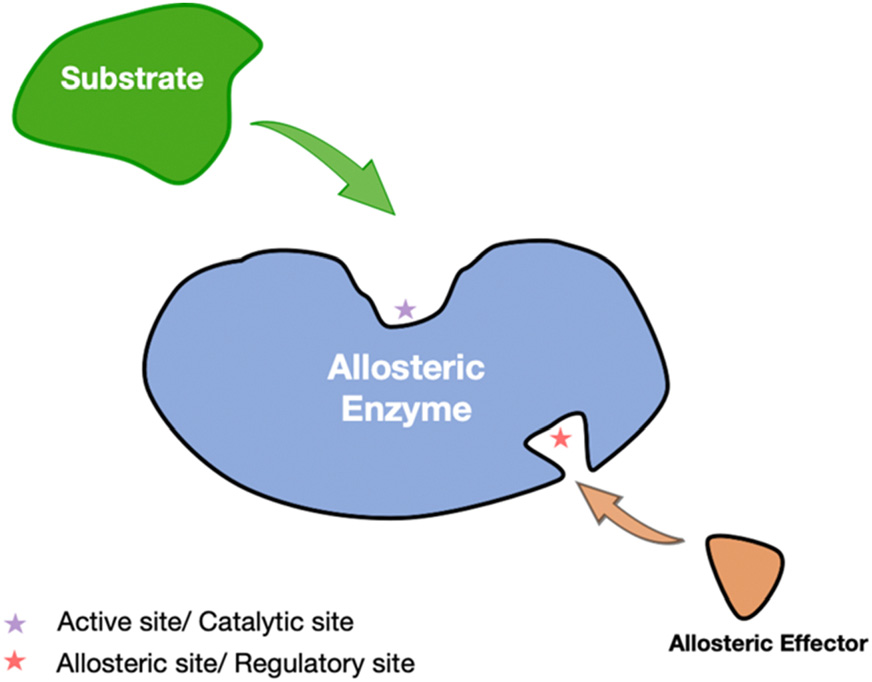
Allosteric regulation in enzymes. The substrate (green) interacts with the active site of the enzyme, while an allosteric modulator (orange) binds to a separate allosteric site (indicated by the red star). This binding event induces conformational changes that modulate the catalytic activity of the enzyme, either enhancing or inhibiting substrate affinity and thereby regulating overall enzymatic function.

Allosteric drugs present distinct advantages over traditional orthosteric drugs, including enhanced specificity and reduced adverse effects. By targeting allosteric sites—typically less conserved across protein families—these drugs allow for selective modulation of specific protein subtypes, offering greater precision in therapeutic interventions. Allosteric regulation, which modulates enzyme activity through conformational changes induced by effector binding at non-active sites, is central to this process (Fig. 1). Additionally, allosteric modulators can act synergistically with orthosteric agents to enhance treatment efficacy, as demonstrated by the combination of GNF-2 and imatinib in the treatment of chronic myelogenous leukemia.^3^ This selective and complementary action underscores the growing appeal of allosteric drugs in contemporary drug development.^4^

## 2. Computational approaches for allosteric site identification

### 2.1 Dynamic and collective motion analysis for allosteric site detection

#### 2.1.1 Molecular dynamics simulations: unveiling dynamic allosteric mechanisms

Molecular dynamics (MD) simulations serve as a powerful computational tool widely employed to investigate the dynamic behavior of biomolecules, including proteins and nucleic acids, at the atomic level.^5^ Based on Newton's law, MD simulations compute interatomic forces and track atomic movements, thereby providing detailed insights into conformational changes and molecular dynamics.^5^ Typically, MD simulations commence with experimentally determined three-dimensional structures, followed by energy minimization and equilibration to approximate physiological conditions. The strength of MD simulations lies in their ability to reveal conformational changes over various timescales, providing dynamic information that is difficult to obtain through traditional experimental methods, especially in the context of enzyme allosteric regulation.

Allosteric regulation refers to the process of modulating enzyme activity through conformational changes, often involving dynamic adjustments in key intermolecular interactions. Since these transitions occur on sub-nanosecond to millisecond timescales, they are challenging to observe directly using traditional experimental techniques. MD simulations, however, provide high temporal resolution, enabling the characterization of regulatory mechanisms. By tracking enzyme conformational changes and internal molecular dynamics, MD simulations facilitate the identification of allosteric sites that govern enzyme activity and signal transduction—information that is often difficult to obtain from static structural analyses alone.^6^

In the study of enzyme allosteric regulation, MD simulations have proven particularly effective in identifying cryptic allosteric sites. For instance, in research on branched-chain α-ketoacid dehydrogenase kinase (BCKDK), static X-ray crystallography failed to reveal certain allosteric sites, whereas MD simulations successfully captured their conformational changes.^7^ By integrating MDpocket algorithms with statistical coupling analysis (SCA) and druggability scoring, researchers further mapped potential druggable allosteric sites.^7^ Similarly, in the study of thrombin, S. Bowerman *et al.* employed MD simulations to analyze the conformational impact of the antagonist hirugen, uncovering cryptic allosteric sites and delineating the underlying dynamic pathways.^8^ Furthermore, Moroni *et al.* utilized MD simulations to investigate the allosteric regulation of mitochondrial Hsp90 (Trap1), revealing that its asymmetric structure plays a critical role in modulating molecular chaperone activity.^9^ Their findings demonstrated how environmental factors induce conformational changes in Trap1, influencing its function, particularly in cancer cells that rely on Trap1 for survival—thus providing a rationale for developing Trap1-targeted therapeutics.

MD simulations have also been instrumental in elucidating the allosteric regulation of membrane-associated proteins. In the study of K-Ras4B, researchers employed MD simulations in conjunction with other computational techniques to investigate its allosteric mechanisms in the membrane-bound state, identifying key sites that regulate GTP-binding activity and interactions with downstream effectors.^10^ Moreover, MD simulations have been applied to various allosterically regulated enzymes, such as LFA-1, p38-α, GR, and MAT2A, revealing crucial dynamic changes that are often overlooked by conventional static experimental methods.^6^ These studies not only enhance our understanding of enzyme function but also provide critical computational insights for structure-based drug design targeting allosteric regulation.

In summary, MD simulations have emerged as a powerful approach for investigating enzyme allosteric regulation, offering dynamic insights beyond the limitations of static structural analyses. As computational power continues to advance and algorithms become more sophisticated, MD simulations will play an increasingly pivotal role in molecular biology and drug discovery, providing essential insights into biomolecular function and the identification of novel therapeutic targets.

##### 2.1.1.1 Enhanced sampling techniques: accelerating the exploration of allosteric sites

MD simulations, when integrated with enhanced sampling techniques and advanced energy analysis, are essential for accurately identifying and characterizing allosteric sites in enzymes.^11–13^ These approaches help overcome the limitations of traditional MD simulations, which may fail to capture rare conformational events critical to allosteric regulation. Enhanced sampling techniques, such as metadynamics and umbrella sampling, accelerate the exploration of conformational space by surpassing energy barriers, thereby revealing hidden allosteric sites that remain inaccessible through conventional MD alone. Collective variable (CV)-based approaches, such as metadynamics (MetaD) and umbrella sampling, are widely employed because they facilitate the exploration of conformational spaces by overcoming energy barriers that can obscure the detection of rare conformational events linked to allosteric regulation. MetaD introduces bias potentials to accelerate sampling along specific CVs, such as those involved in allosteric transitions or effector binding events. By applying a time-dependent bias to the CV space, MetaD enables the system to escape local energy minima, thereby facilitating the reconstruction of the free energy surface and revealing new conformational states where potential allosteric sites may emerge. Variational Enhanced Sampling (VES) further refines this approach by optimizing a function to determine the optimal bias potential, promoting more efficient exploration of the free energy landscape and aiding in the identification of cryptic allosteric sites.^11^ Umbrella sampling, another CV-based method, introduces harmonic potentials to guide sampling toward regions where allosteric sites are likely to form.^14^ By dividing the conformational space into windows along a selected CV, umbrella sampling overcomes energy barriers and facilitates the convergence of free energy calculations, thereby uncovering hidden conformations and transition states associated with allosteric regulation.

When the identification of suitable CVs is challenging, accelerated MD (aMD), replica exchange MD (REMD), and Steered MD (SMD) become invaluable. The aMD modifies the potential energy surface by introducing a boost potential, allowing the system to cross high energy barriers and explore a broader conformational space.^15^ This approach can capture millisecond-timescale events within hundreds of nanoseconds of simulation, effectively revealing transient allosteric pockets that would otherwise remain inaccessible. REMD involves simulating multiple replicas of the enzyme at different temperatures, with periodic exchanges between replicas to facilitate conformational transitions.^16^ This multiscale sampling technique enables the system to overcome energy barriers and explore a wider range of conformational states, aiding in the discovery of allosteric sites hidden in high-energy conformations and providing deeper insights into the functional dynamics of enzymes. SMD offers a complementary approach by applying an external force to the system along a predefined pathway, often by “pulling” specific atoms or molecules through the conformational space.^17^ Inspired by experimental techniques such as atomic force microscopy, SMD drives the system from one state to another, exploring transitions that may reveal hidden allosteric sites or provide insight into the pathways leading to allosteric regulation.^18^ By applying an external bias potential, SMD probes the free energy landscape and identifies key regions associated with allosteric transitions, offering a detailed mechanistic understanding of enzyme dynamics.^19^

Beyond CV-based methods, non-Boltzmann sampling techniques such as multicanonical sampling, entropy sampling, and Wang–Landau sampling adjust sampling probabilities to uniformly explore the energy landscape of enzymes without requiring prior knowledge of allosteric transitions.^20–22^ Through the use of these methods, it is possible to enhance the detection of rare conformations by reweighting the exploration of system conformational space. The Wang–Landau sampling, in particular, constructs iterative estimates of the density of states, enabling a more uniform sampling process and thereby uncovering elusive allosteric sites.^23^ Free energy calculations play a pivotal role in elucidating the thermodynamics underlying allosteric site formation, offering critical insights into the stability and feasibility of these sites for drug targeting.^24^ Methods such as thermodynamic perturbation, thermodynamic integration, and non-equilibrium approaches provide insights into the stability and feasibility of these sites.^25^ By calculating changes in energy between different conformations, thermodynamic perturbation estimates differences in free energy. Alternatively, thermodynamic integration gradually alters the system state variables to compute changes in free energy along pathways that may involve the opening or closing of allosteric sites.^26^ Non-equilibrium methods, such as the Jarzynski equality, estimate equilibrium free energy differences by analyzing work distributions from non-equilibrium processes, offering a detailed view of the energetic landscape associated with allosteric site formation.^27^ By integrating these advanced sampling techniques with MD simulations, researchers can more effectively identify and characterize allosteric sites in enzymes.^28^ This comprehensive approach offers a deeper understanding of the structural and dynamic features underlying allosteric regulation, providing novel insights into potential therapeutic targets for modulating enzyme activity through allosteric mechanisms. The combination of these methodologies enables a more nuanced exploration of the dynamic landscape of enzyme, capturing the transient and often elusive nature of allosteric sites that are crucial for understanding and manipulating enzyme function.

##### 2.1.1.2 Structural analysis methods: integrating MD simulations for allosteric site identification

In addition to MD simulations and enhanced sampling techniques, structural analysis methods are crucial for identifying allosteric sites, particularly when they are not apparent in static structures. Several energy-based computational tools, Q-SiteFinder,^29^ FTMap,^30^ and its web-based extension FTMove^31^ are prominent tools based on energy analysis (Table 1). have been prominent in the identification of ligand-binding hotspots. These methodologies can be effectively coupled with MD simulations to refine the identification of allosteric sites by incorporating protein conformational dynamics.

**Table 1:** Methodologies for Methodologies for computational allosteric site identification and prediction

Name	Ref	Web server available	Methods and applications
AlphaFold2	32	https://alphafoldserver.com/	High-accuracy 3D protein structure prediction based on sequence data, useful for allosteric site prediction
AlloPred	33	No	Predicts allosteric sites using a combination of structural and evolutionary features
AlloReverse	34	https://mdl.shsmu.edu.cn/AlloReverse/	Predicts allosteric communication based on reversed allosteric communication theory
AlloSigMA 2	35	https://allosigma.bii.a-star.edu.sg/home/	Analyzes allosteric signal propagation to assess effects induced by ligand binding or mutations
Allosite	36	https://mdl.shsmu.edu.cn/AST/	Uses support vector machines (SVM) to predict allosteric sites, applied in protein allosteric regulation analysis
AllosES	37	No	Integrates sequence entropy and evolutionary conservation to identify allosteric sites
ConSeq	38	https://conseq.bioinfo.tau.ac.il/	Identifies functionally important regions in proteins based on sequence conservation without requiring 3D structures
ConSurf	39	https://consurf.tau.ac.il/consurf_index.php	Evolutionary conservation analysis of proteins to identify functional residues based on multiple sequence alignments
ConSurf-DB	40	https://consurfdb.tau.ac.il/	A database for evolutionary conservation scores of proteins
FTMap	41	https://ftmap.bu.edu/	Identifies ligand-binding hotspots by distributing small organic probes over protein surfaces
FTMove	31	https://ftmove.bu.edu/	Analyzes multiple conformations to identify dynamic binding hotspots across different protein structures
Fpocket	42	https://durrantlab.pitt.edu/fpocketweb/	Geometric analysis for pocket detection and allosteric site identification on protein surfaces
GHECOM	43	https://pdbj.org/ghecom/	Uses mathematical morphology to reveal complex hidden pockets on protein surfaces
KeyAlloSite	44	No	Predicts key allosteric residues using the evolutionary coupling model (ECM), particularly for long-range interactions
Minimotif Miner	45	https://mnm.engr.uconn.edu	Searches for short functional motifs in protein sequences to reveal functional and evolutionary insights
PASSer	46	https://passer.smu.edu/	Predicts allosteric sites using machine learning (ML), leveraging geometric and topological features
PocketMiner	47	https://pocketminer.azurewebsites.net/	Graph neural network (GNN)-based method for allosteric site prediction, effectively processing large datasets
Q-SiteFinder	29	https://www.modelling.leeds.ac.uk/qsitefinder	Detects binding sites using van der Waals probes to map favorable binding regions
SBSMMA	48	No	A statistical mechanics model that simulates ligand binding effects for allosteric site identification
SURFNET	49	No	Geometric pocket detection based on 3D contour generation, identifies large binding pockets

FTMap^41^ facilitates the identification of potential allosteric regions by mapping the binding of small organic probe molecules across the enzyme surface, thus delineating energetically favorable binding pockets. Similarly, Q-SiteFinder determines ligand-binding sites by computing interaction energies between the protein and a van der Waals probe, highlighting energetically privileged regions for ligand interactions.^29^ However, these tools traditionally focus on single static protein structures. To overcome this limitation, FTMove extends the capability of FTMap by incorporating ensemble-based structural analyses, leveraging multiple protein conformations derived from experimentally resolved structures in the Protein Data Bank (PDB) or MD simulations. By systematically mapping allosteric hotspots across a diverse set of conformational states, FTMove enables a more dynamic characterization of allosteric sites, thus providing insights into the structural plasticity of binding pockets. This approach is particularly advantageous for detecting cryptic allosteric sites, which only become transiently accessible during specific conformational states observed in MD simulations. In a study introducing FTMove, the tool successfully identified binding sites in 22 proteins with known allosteric sites, elucidating the structural mechanisms underlying the formation of transient binding pockets and conformationally dynamic allosteric regulation.^31^ By integrating MD-generated structural ensembles, FTMove can provide a more physiologically relevant approach for allosteric site prediction, advancing both rational drug design and structural biology research.

Complementing energy-based methods like FTMap,^41^ Q-SiteFinder,^29^ FTMove^31^ and AlloSigMA 2,^35^ several geometry-based tools offer a complementary strategy for refining allosteric pocket prediction and can be seamlessly integrated with MD simulations to improve accuracy.^29,31,41^ Among these tools, Fpocket has been widely employed for its efficiency in rapidly detecting binding pockets through the analysis of surface topology, cavity depth, and hydrophobicity patterns.^42^ Its capability to analyze multiple conformational states makes it particularly well-suited for integration with MD simulations, enabling the identification of dynamically accessible allosteric sites that are not observable in static structures. Notably, Fpocket has successfully identified allosteric sites in enzymes such as Uridylate Kinase, uncovering a GTP-binding central cavity, as well as in Pyruvate Kinase M2, where it predicted a regulatory pocket linked to tumor suppression.^50^ Additional geometric pocket-detection tools, including LIGSITEcsc, which integrates solvent contact analysis with residue conservation scoring,^51^ and CASTp, which provides comprehensive geometric descriptors such as pocket volume and solvent-accessible surface area, serve as valuable enhancements when coupled with MD-derived conformational ensembles.^52^ Similarly, SURFNET,^49^ using three-dimensional contour generation, excels in identifying large binding pockets, while GHECOM^43^ employs mathematical morphology to reveal complex, hidden pockets.

The integration of MD simulations with both energy-based and geometry-based computational methodologies establishes a robust and multi-faceted framework for allosteric pocket identification. This synergistic approach not only enhances the predictive accuracy of computational models but also provides structural insights into allosteric mechanisms, thereby facilitating the rational design of allosteric modulators and contributing to the broader field of computational drug discovery.

#### 2.1.2 Normal mode analysis: identifying large-scale conformational changes

Normal mode analysis (NMA) is a valuable computational method for investigating protein dynamics, particularly in identifying large-scale collective motions.^53^ However, its reliance on harmonic approximations limits its ability to model non-linear dynamic processes, which may reduce its accuracy in complex biological systems. NMA typically relies on the elastic network model (ENM), which simplifies protein structures by representing residues as nodes connected by harmonic springs, allowing efficient calculation of low-frequency vibrational modes. These modes often correspond to large conformational changes between different protein states and are crucial for understanding protein flexibility and global motions.^54^

A notable application of NMA is its effectiveness in predicting the global dynamic behavior of enzymes and identifying their active sites. For example, the EXPOSITE technique uses NMA to capture the open-close movements of enzymes in low-frequency vibrational modes and analyze solvent accessibility changes during these movements, particularly around the active site residues.^55^ By calculating the solvent accessibility changes of different pocket regions during dynamic deformation, EXPOSITE accurately predicts the locations of active sites and ranks these pockets accordingly. Unlike traditional methods that rely on static geometric features, EXPOSITE integrates dynamic exposure changes, significantly improving prediction accuracy across multiple enzyme datasets. This example illustrates the potential of NMA in identifying enzyme active sites, uncovering protein functions, and guiding drug design. Compared to MD simulations, NMA offers a significant computational efficiency advantage. Although MD can capture detailed time evolution and microscopic motions of proteins, its high computational cost makes it challenging to perform simulations over large time scales and systems.^56,57^ In contrast, NMA approximates the potential energy surface of proteins as a harmonic potential, enabling the identification of low-frequency vibrational modes associated with large-scale conformational changes, which are often key to biological functions.^58^ For instance, in the study of lysozyme, NMA revealed hinge-bending movements between its domains, demonstrating how the protein adapts its conformation through low-frequency modes to accommodate substrate binding.^59^ With the help of this low-frequency mode, the function of lysozyme can be explained, and the ability of NMA to capture critical flexible regions related to the function of proteins is further validated. Despite certain limitations, such as its reliance on harmonic approximations and exclusion of solvent effects, NMA remains a valuable tool in molecular docking and structural analysis, particularly when protein flexibility significantly impacts ligand-binding predictions. Techniques like EXPOSITE demonstrate how incorporating dynamic exposure changes can significantly improve the prediction of active sites. As a result, NMA complements MD simulations by providing insights into protein dynamics and functional mechanisms, proving useful in fields such as drug discovery and enzyme regulation. For example, perturbation response scanning (PRS) is an effective computational method that combines the ENM with linear response theory (LRT) to explore allosteric sites in proteins, often in conjunction with MD simulations.^60^ Techniques like PRS integrate NMA with linear response theory to explore allosteric sites in enzymes. PRS systematically applies random perturbative forces to each residue within the protein structure and calculates the overall response to these perturbations, identifying key residues that induce global conformational changes.^61^ For instance, Paul *et al.* integrated PRS with MD simulations to investigate the dynamic allosteric regulation between the main proteases of SARS-CoV-1 and SARS-CoV-2.^62^ They performed MD simulations on multiple structures of both proteases using the AMBER software package and used PRS to calculate the dynamic flexibility index (DFI) and dynamic coupling index (DCI), providing an in-depth analysis of the dynamic coupling between different residues. Their study revealed that the catalytic sites of SARS-CoV-2 (*e.g.*, H41 and C145) exhibit significantly enhanced inter-chain dynamic coupling with other regions of the protein, particularly residues on chain B (*e.g.*, E55, I59, R60, N277, R279, and L286), a feature absent in SARS-CoV-1.^62^ Further analysis indicated that the dynamic changes in SARS-CoV-2 primarily occur in regions distant from the mutation sites, specifically at the dimer interface and areas critical for enzymatic activity regulation.^62^ Additionally, the study found that key allosteric sites in SARS-CoV-2 exhibit behavior opposite to that in SARS-CoV-1 upon inhibitor binding. These findings elucidate the complex mechanisms of dynamic regulation in SARS-CoV-2 and provide new targets and strategies for antiviral drug development.

In summary, the integration of MD simulations, enhanced sampling techniques, energy-based structural analysis methods like FTMap,^41^ Q-SiteFinder,^29^ and FTMove,^31^ structure-based tools such as Fpocket^42^ and others, and computational methods like NMA offers a comprehensive framework for identifying and characterizing allosteric sites in enzymes (Table 1). This multidimensional approach allows for a deeper understanding of the structural and dynamic features underlying allosteric regulation, providing valuable insights for drug discovery and the modulation of enzyme activity.

### 2.2 Evolutionary dynamics and sequence-based methods for predicting allosteric sites

One of the most effective strategies for identifying allosteric sites in enzymes is through evolutionary and sequence-based methods. By analyzing the evolutionary history and sequence variations of enzymes, these approaches can pinpoint potential allosteric sites, particularly those that are distal from the active site but play a critical role in enzyme regulation. Below are several commonly employed evolutionary and sequence analysis techniques specifically designed for uncovering allosteric sites in enzymes.

#### 2.2.1 Evolutionary conservation analysis: identifying key functional residues in allosteric sites

Evolutionary conservation analysis is a crucial tool for identifying key functional sites, particularly allosteric sites, in proteins. By comparing homologous proteins or genes across different species, researchers can uncover residues or structural regions that have been highly conserved throughout evolution, which often play critical roles in regulating protein function, especially in allosteric mechanisms. Although allosteric sites are often located far from the active center, they can influence protein function by modulating conformational changes. As protein sequences undergo variations (*e.g.*, mutations, insertions, or deletions), these allosteric communication pathways diversify during evolution to adapt to different functional requirements.^63^ Additionally, evolutionary conservation analysis can be conducted using tools such as ConSurf,^39^ ConSurf-DB,^40^ ConSeq,^38^ or Minimotif Miner,^64^ which can map conservation scores without the three-dimensional structure of enzymes, aiding in the identification of potential allosteric sites (Table 1).

Frlan *et al.* employed multiple sequence alignment (MSA) and phylogenetic tree construction to analyze protein sequences of seven enzymes from pathogenic bacteria, which were obtained from the UniProt database.^65^ They utilized the ConSurf tool and SiteMap to calculate conservation scores for the amino acid residues of these enzymes, assigning scores from 1 to 9, with higher scores indicating greater conservation across species.^39,65^ These scores were then mapped onto the three-dimensional crystal structures of the enzymes, visually highlighting regions with high functional conservation. Their analysis revealed that most of the substrate-binding sites in the enzymes, particularly in four key enzymes of the shikimate pathway (DHQS, SDH, EPSPS, and CS), exhibited high conservation, overlapping with functional sites. This indicates that these regions are critical for bacterial survival and represent potential targets for broad-spectrum antimicrobial drugs. However, the allosteric sites of some enzymes, such as DAHPS, showed higher variability across species, limiting their potential as broad drug targets. Additionally, the study revealed that while some binding sites are highly conserved, their polar or charged nature may make it difficult to identify drugs with strong binding affinity. By integrating evolutionary conservation analysis with druggability assessment, Frlan *et al.* identified the most suitable binding sites in the shikimate pathway for developing broad-spectrum antimicrobial drugs.^65^

As in the study of Frlan, Leander *et al.* employed evolutionary conservation analysis to identify allosteric sites within the TetR protein. Through multiple sequence alignments, they found that allosteric sites in TetR exhibit lower evolutionary conservation compared to structural stability sites, yet these less-conserved residues still play key roles in allosteric regulation.^66^ Leander *et al.* employed deep mutational scanning and MD simulations to reveal how distal residues, despite their lower conservation, can restore function through long-range thermodynamic modulation.^66^ For example, residues R49 and N129 in the α4 and α9 helices, though not highly conserved in evolution, were identified as critical components of the allosteric network, exhibiting significant functional flexibility.^66^ This decoupling between evolutionary conservation and function suggests that while allosteric sites may not be highly conserved, they can still regulate protein function through multiple mechanisms, offering important implications for drug design.

The combined findings of Frlan *et al.* and Leander *et al.* highlight the value of evolutionary conservation analysis in identifying both allosteric sites and druggable targets. By leveraging multiple sequence alignments, conservation analysis tools like ConSurf, and integrating functional and structural data, researchers can identify residues or regions that are functionally critical and explore their potential as drug targets.^39,40^ Evolutionary conservation analysis not only reveals the value of highly conserved substrate-binding sites for drug development but also demonstrates that, despite lower conservation, allosteric sites play a pivotal role in regulating protein function, showcasing the broad applications of this tool in biology and drug discovery.

#### 2.2.2 Co-evolutionary analysis: uncovering long-range residue interactions for allosteric regulation

Co-evolutionary analysis is a key tool for predicting protein interactions and regulatory functions, with broad applications in bioinformatics and structural biology.^67^ Currently, mutual information (MI),^68^ statistical coupling analysis (SCA),^69^ and direct coupling analysis (DCA)^70^ are the three main methods used in co-evolutionary analysis, each demonstrating distinct advantages and limitations depending on the research context. MI reveals evolutionary coupling relationships by assessing the shared information between pairs of amino acids.^68^ While it is computationally simple and can rapidly identify residue dependencies, it is susceptible to background noise, leading to a high false positive rate. In contrast, SCA identifies co-evolving residues based on mutational patterns in multiple sequence alignments, making it particularly suitable for detecting long-range interactions.^69^ However, its predictive performance is highly dependent on the quality and quantity of homologous sequences. DCA, by constructing a maximum entropy model to filter out indirect coupling signals, can more precisely predict directly coupled residue pairs, excelling in local interaction and structural prediction tasks.^70^

DCA has demonstrated exceptional accuracy in predicting local protein interactions. Fantini *et al.* showed that DCA could accurately capture the structural features of CyaY, a protein involved in iron–sulfur cluster biosynthesis, and elucidate the dimerization mechanism of IscU and its coordination with FeS clusters.^71^ Additionally, DCA successfully predicted the local interactions between IscU and IscS, further validating its effectiveness in short-range interaction predictions. However, DCA exhibits certain limitations in predicting long-range cooperative effects. Bravi *et al.* found that DCA struggles to accurately capture long-distance interactions between distant epitopes, which are critical in regulating allosteric functions.^72^ To address this issue, Bravi and colleagues proposed a neural network-based nonlinear model that better captures complex long-range cooperative effects, particularly in allosteric proteins involving multiple structural regions.

La Sala *et al.* applied SCA to identify co-evolving amino acids in proteins to uncover allosteric regulatory mechanisms.^7^ By calculating the coverage score (CS) of co-evolving residues in allosteric pockets, they evaluated the functional significance of these pockets. Their results showed that the known allosteric pockets in GR, BCKDK, and p38-α had high CS values, indicating that the residues within these pockets co-evolved during evolution, contributing to the transmission of allosteric signals. As a result, SCA is limited in its ability to predict sequence homologies in cases where the quality of homologous sequence data is inadequate, as in the case of MAT2A and LFA-1 sequences. Nevertheless, SCA revealed networks of long-range co-evolving residues, which are critical for understanding allosteric regulatory mechanisms. To improve predictive accuracy, La Sala *et al.* combined SCA with druggability score analysis and rigidity analysis, constructing a three-parameter model that significantly enhanced the identification of allosteric pockets.^7^

In recent years, co-evolutionary computational methods have made significant progress in identifying key allosteric residues (allo-residues) in proteins. Xie *et al.* developed the KeyAlloSite method, which uses the Evolutionary Coupling Model (ECM) to successfully predict key allosteric residues in several proteins and reveal strong coupling relationships between these residues and orthosteric site residues.^44^ KeyAlloSite excels in predicting cancer-related mutation sites and residues distant from catalytic sites but essential for enzymatic function. It provides a systematic and efficient tool for allosteric drug design and protein engineering, addressing the flat structure–activity relationship problem commonly encountered in the optimization of allosteric molecules and advancing the development of allosteric drugs and the design and optimization of functional proteins.

In summary, MI, SCA, and DCA each offer unique advantages in co-evolutionary analysis, and their integration with emerging tools such as neural network models and KeyAlloSite can more effectively predict protein functions, structures, and interactions.^44,68–70^

#### 2.2.3 Sequence entropy analysis: quantifying variability and information flow in allosteric networks

Sequence entropy analysis is a computational method used to quantify the variability and information content within protein or nucleotide sequences.^73^ By measuring the degree of disorder or uncertainty in the arrangement of residues, this approach helps identify regions of high or low variability within the sequence.^74^ In proteins, regions with high sequence entropy typically correspond to flexible or functionally diverse areas. In contrast, low entropy regions indicate conserved areas critical for structural stability or function, such as active or binding sites.

In allosteric regulation, sequence entropy analysis is crucial for revealing the evolutionary conservation and functional relevance of residues that drive allosteric behavior. It offers insights into allosteric mechanisms by highlighting residues that contribute to dynamic communication networks within the protein. Transfer entropy, in particular, serves as an important metric by capturing time-delayed correlations between residues, thereby mapping the information transfer between different sites within a protein. By quantifying how the dynamic behavior of one site affects another distant site, transfer entropy provides a unique means to identify key residues involved in allosteric regulation. For example, in the study of the allosteric mechanism of biotin protein ligase, transfer entropy analysis revealed certain residues with high transfer entropy values, suggesting their roles as mediators of allosteric communication.^37^ This method complements traditional structural analysis by providing a more robust framework for studying the complexity of allosteric systems, especially in cases where conventional approaches are insufficient to identify allosteric sites.

Cecconi *et al.* employ sequence entropy analysis to explore allosteric mechanisms within the protein ubiquitin.^75^ This method, particularly transfer entropy, quantifies the flow of information between residues, offering insights into the directional relationships that drive allosteric regulation.^75^ Unlike traditional correlation analysis, which captures coordinated fluctuations between residues without inferring causality, sequence entropy analysis offers a way to distinguish between mere correlations and true causal influences. An essential part of sequence entropy analysis is transfer entropy, which measures how knowing the state of one residue reduces the uncertainty about the future state of another residue.^74^ This approach reveals the roles of specific residues in ubiquitin as information donors or acceptors, elucidating the flow of allosteric signals across the protein. By utilizing sequence entropy analysis, researchers can determine how perturbations in one part of the protein lead to functional changes at distant sites, thereby mapping the allosteric pathways that regulate the activity of ubiquitin. The study also shows that residues involved in allosteric control of ubiquitin can be linked through transfer entropy, emphasizing their roles in modulating interactions with ubiquitinase. Cecconi *et al.* leverage sequence entropy analysis to provide a detailed understanding of dynamic allosteric networks in proteins, highlighting the utility of this method in identifying key regulatory residues and pathways.^75^

Furthermore, integrating entropy-based methods with evolutionary data, such as spatial proximity evolutionary scores, enhances the accuracy of identifying allosteric modulators by considering both dynamic and structural constraints. By combining sequence entropy with evolutionary analysis, tools like AllosES have demonstrated exceptional predictive performance in identifying allosteric sites, showcasing the extensive potential of this approach in understanding and targeting allosteric regulation in proteins.^37^

### 2.3 Graph-theory and machine learning-based approaches

#### 2.3.1 Graph-based methods for allosteric site prediction

Graph theory approaches offer novel perspectives in enzyme research. Enzymes can be represented as networks of amino acids, where edges correspond to interactions between residues. Network analysis methods such as AlloSite^36^ and AlloPred^33^ can identify key residues that act as communication hubs within the network, these residues often correspond to allosteric sites of the enzyme (Table 1). By unveiling the topological connections among amino acids, these methods enhance our understanding of the regulatory mechanisms within enzymes and provide a crucial theoretical foundation for the identification and prediction of allosteric sites. This graph theory-based network analysis not only aids in exploring the global dynamics of proteins but also effectively screens potential drug targets, thereby opening new avenues for drug design.

Allosite is a computational tool designed to predict allosteric sites in proteins, playing a crucial role in drug discovery due to the advantages of targeting allosteric sites, including higher specificity, fewer side effects, and lower toxicity compared to orthosteric drugs.^36^ The method integrates pocket-based analysis and support vector machine (SVM) algorithms to accurately identify potential allosteric sites. Allosteric sites are regions distinct from the active site, where ligand binding can induce conformational changes that modulate the function of prteins. Given their lower evolutionary conservation, allosteric sites present a more selective target for therapeutic interventions. The Allosite model is trained on high-quality datasets from the allosteric database (ASD)^76^ and has been validated with cross-validation, demonstrating over 95% accuracy.^36^ In the study conducted by Wenkang Huang and colleagues, the Allosite tool was successfully employed to identify allosteric sites in various proteins.^4^ By extracting non-redundant allosteric protein-modulator co-crystal structures from the allosteric database, the research team used a support vector machine model to predict allosteric sites, achieving successful outcomes across several proteins. For instance, in the study of Bcr-Abl kinase, Allosite accurately identified an allosteric site at the myristate-binding site of Bcr-Abl, where the allosteric modulator GNF-2 binds, effectively regulating the activity of proteins. Additionally, Allosite was able to rapidly and precisely identify 0-4 potential allosteric sites in other proteins, providing valuable targets for further drug development efforts.^4^

Solvent mapping is a computational technique widely used to identify potential binding pockets, including allosteric sites, on protein surfaces by simulating interactions between small probe molecules and the protein surface, thereby determining energetically favorable regions.^48^ These hotspots, characterized by frequent probe binding, indicate areas of high ligand-binding affinity, making them ideal candidates for drug discovery, particularly for targeting allosteric regulation, where binding at distant sites modulates enzyme activity. Complementing this, ENM offers a valuable approach for analyzing how local perturbations, such as ligand binding, induce global conformational changes in proteins.^77^ By modeling proteins as networks of nodes connected by springs, ENM predicts low-frequency motions linked to functional shifts and helps identify key residues involved in allosteric signaling. This combined approach enhances the understanding of protein dynamics and informs the design of allosteric modulators. For instance, Ayyildiz and colleagues utilized solvent mapping, ENM, and sequence/structural alignments to investigate allosteric sites in glycolytic enzymes, including phosphofructokinase (PFK), glyceraldehyde-3-phosphate dehydrogenase (GADPH), and pyruvate kinase (PK).^78^ Their research identified several allosteric sites at subunit interfaces, with ENM revealing their impact on global enzyme dynamics. Furthermore, sequence alignments indicated low conservation of these sites across bacterial, parasitic, and human species, highlighting their potential as species-specific drug targets.

#### 2.3.2 Machine learning and AI-based methods for rapid prediction of cryptic allosteric site

Graph neural networks (GNNs) have proven to be an effective method for predicting cryptic allosteric sites in enzymes. These models represent molecular structures as graphs, with atoms functioning as nodes and chemical bonds as edges, enabling the network to learn complex atomic interactions through graph convolution operations.^79^ By propagating information through these graph structures, GNNs can extract local structural features and predict potential allosteric sites. One prominent example is PocketMiner, a model built on the geometric vector perceptron (GVP) architecture.^47^ This model accurately predicts cryptic allosteric sites by encoding protein residues and their spatial relationships into a graph format, integrating this information across multiple layers of the network. PocketMiner offers a significant advantage over traditional MD simulations due to its rapid prediction capability and high accuracy, making it well-suited for large-scale protein screenings and diverse datasets.^47^ In applied studies, PocketMiner has successfully identified cryptic allosteric sites in several enzymes, demonstrating its value in drug discovery and allosteric regulation research. For example, in the study of PIM2, a serine/threonine kinase involved in cancer, PocketMiner predicted a cryptic allosteric site located near the orthosteric site, which was subsequently validated through MD simulations.^47^ This discovery suggests a new potential target for allosteric regulation in PIM2. Similarly, PocketMiner identified cryptic allosteric sites in the WNT2 protein, despite the lack of visible binding sites in its ground-state structure.^47^

PASSer is an advanced tool designed for the rapid and accurate prediction of protein allosteric sites, which play a crucial role in regulating protein function through conformational changes induced by ligand binding at sites distal to the active site^46^ (Table 1). The tool integrates ensemble learning techniques, including eXtreme Gradient Boosting (XGBoost) and graph convolutional neural networks (GCNNs), to extract and analyze the geometric and physicochemical properties of protein pockets identified by the FPocket algorithm.^80^ These machine learning (ML) models enable PASSer to accurately predict allosteric sites by leveraging structural features and topological data. The tool has demonstrated high performance in identifying potential allosteric sites by leveraging both the geometric and topological features of protein structures. PASSer also employs automated machine learning (AutoML) techniques, which streamline the process of model selection and hyperparameter tuning, significantly enhancing both the efficiency and accuracy of predictions.^81^ A notable feature of PASSer is its use of ranking algorithms, including LambdaRank, which prioritize potential allosteric pockets based on their likelihood of functional relevance, thus improving the interpretability of its predictions.^46^ PASSer was employed to predict the allosteric sites in the light-oxygen-voltage (LOV) domain protein^46^ In using ensemble learning models, including extreme gradient boosting (XGBoost)^82^ and Graph. Convolutional neural networks (GCNNs),^80,83^ PASSer successfully identified the top-ranked allosteric pocket with 89.65% probability.^80^ According to these results, the tool was able to accurately predict allosteric sites that were experimentally validated, demonstrating its robust performance for allosteric site prediction.

**Fig. 2:**
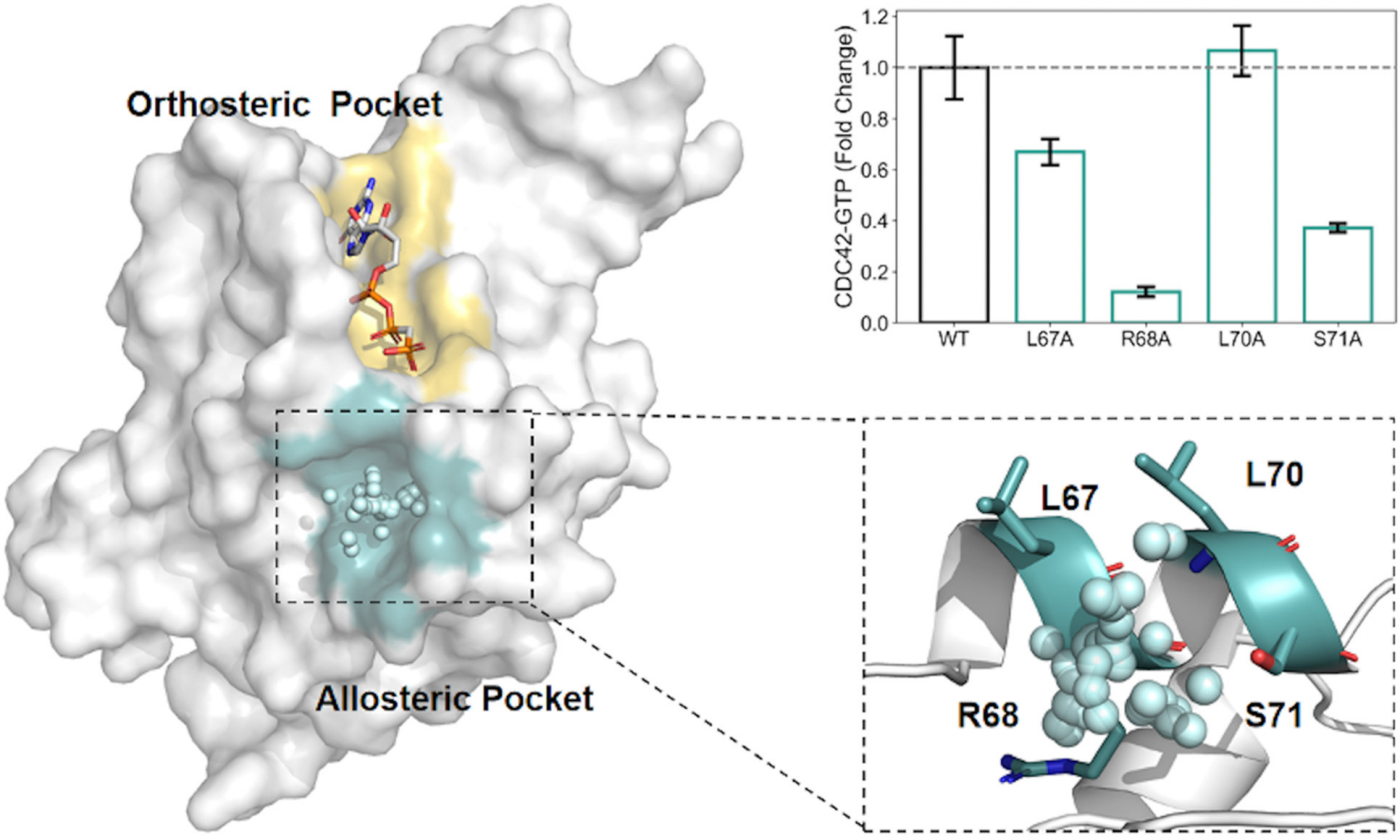
Structural and functional analysis of the allosteric modulation identified by AlloReverse. (A) The enzyme structure highlights the orthosteric pocket (yellow) and the allosteric pocket (blue), which was computationally identified using the AlloReverse approach. The key allosteric residues (L67, R68, L70, and S71) within the allosteric pocket are shown in their spatial context, interacting with the modulator. (B) Mutational analysis of these residues demonstrates their effects on CDC42-GTP binding activity, expressed as fold change relative to the wild-type (WT). The R68A mutation causes a substantial decrease in activity, underscoring its critical role in allosteric regulation, whereas L67A and L70A show moderate effects.

AlloReverse is an advanced computational tool designed to predict and analyze allosteric sites in enzymes using the reversed allosteric communication theory, which posits bidirectional regulation between allosteric and orthosteric sites^34^ (Fig. 2). By integrating protein dynamics with ML, AlloReverse offers comprehensive predictions of allosteric residues, sites, and regulatory pathways, providing a valuable resource for understanding enzyme regulation and aiding in allosteric drug design. A notable application of AlloReverse is its use in studying the enzyme CDC42, a GTPase involved in cytoskeletal regulation. AlloReverse predicted a previously unknown allosteric site near the orthosteric GTP-binding region.^34^ Experimental validation through site-directed mutagenesis of key residues, such as L67, R68, and S71, demonstrated a significant decrease in GTP binding, confirming the functional importance of this allosteric site.

AlphaFold, one of the most advanced AI models, enables high-precision prediction of protein three-dimensional structures from sequence data.^84^ It is a key tool in predicting allosteric sites, which has become a leading trend in current research. The AlloMAPS2 program, for example, uses a structure-based statistical mechanics model (SBSMMA) to quantify allosteric communication within proteins, integrating AlphaFold and Pfam-trRosetta predictions.^85^ To quantify the allosteric effects of mutations and small molecule probes on individual residues, the model creates allosteric signalling maps (ASMs) and allosteric probing maps (APMs).^85^ A mathematical model reveals how structural changes, such as mutations or small molecule binding, influence allosteric sites, enabling AI-predicted structures to be identified quickly and accurately with potential allosteric sites. AlloMAPS 2^85^ has been successfully applied across multiple studies, including research on the SARS-CoV-2 spike protein, where AlphaFold-predicted structures were analyzed using ASMs to predict how distal mutations influence the dynamics of the receptor-binding domain through allosteric mechanisms.^86^ Additionally, APMs have been used to simulate small molecule binding, successfully identifying potential allosteric targets, thus providing valuable insights for drug development. Simultaneously, Casadevall *et al.* utilized AlphaFold2, in conjunction with deep learning, MD simulations, and other computational methods, to investigate conformational changes in enzymes and active site pockets for both orthosteric and allosteric sites^32^ (Table 1).

Simultaneously, the latest advancements in ML-based allosteric site prediction increasingly emphasize the importance of integrating dynamic molecular features to enhance predictive accuracy. Recent studies have begun incorporating MD simulations to extract conformational flexibility, residue interaction networks, and ligand-induced dynamic changes, thereby improving the identification of allosteric regulatory sites. A recent study by Frasnetti *et al.* leveraged the combination of MD simulations and ML algorithms to predict the functional characteristics of kinase ligands.^87^ They employed long-timescale MD simulations to capture ligand-induced conformational changes in cyclin-dependent kinases (CDKs) and utilized random forest (RF), support vector machine (SVM), and multilayer perceptron (MLP) to classify ligands as orthosteric or allosteric binders. The results demonstrated that RF achieved the highest classification accuracy of 91%, outperforming other models. This approach was further validated by correctly classifying several FDA-approved CDK inhibitors, including Palbociclib and Abemaciclib, as orthosteric binders. This study highlights the potential of integrating MD-derived dynamic features with ML-driven classification models to improve the accuracy and reliability of allosteric site prediction. With the continuous advancement of computational methodologies, the integration of MD simulations and ML algorithms is expected to play a pivotal role in predicting cryptic allosteric sites, characterizing ligand-binding mechanisms, and optimizing allosteric drug design strategies. By leveraging dynamic molecular data, these approaches provide new insights into enzymatic allosteric regulation and contribute to the accelerated discovery of novel therapeutic.

## 3. Case studies: exploring enzymatic allosteric modulators through computational approaches

### 3.1 Computational approaches in identifying Sirtuin 6 (SIRT6) allosteric modulators

Sirtuin 6 (SIRT6), an NAD+-dependent deacetylase, plays a critical role in regulating DNA repair, metabolism, inflammation, and genomic stability.^88–90^ By deacetylating histones such as H3 at lysine 9 (H3K9ac) and lysine 56 (H3K56ac), SIRT6 maintains chromatin integrity and facilitates DNA damage repair.^91–93^ Additionally, SIRT6 suppresses transcription in centromeric regions through deacetylation of H3K18ac, preventing chromosomal missegregation.^93^ The enzymatic activity of SIRT6 is closely linked to intracellular NAD^+^ levels, influencing the cellular energy state. SIRT6 is also involved in regulating cellular metabolism, acting through the AMPK pathway to promote glucose and fatty acid metabolism, ensuring cellular energy homeostasis.^94^ Furthermore, SIRT6 exerts anti-inflammatory effects by deacetylating and inhibiting nuclear factor κB (NF-κB), thereby reducing the production of pro-inflammatory cytokines. In cancer biology, SIRT6 primarily acts as a tumor suppressor by inhibiting oncogenes such as c-Myc, HIF-1α, and c-Jun, modulating key signaling pathways like PI3K/Akt and MAPK/ERK, and inducing cell cycle arrest and apoptosis.^95^ However, the role of SIRT6 in cancer is highly complex and context-dependent, necessitating a deeper understanding of its regulation to develop targeted therapies.

**Fig. 3:**
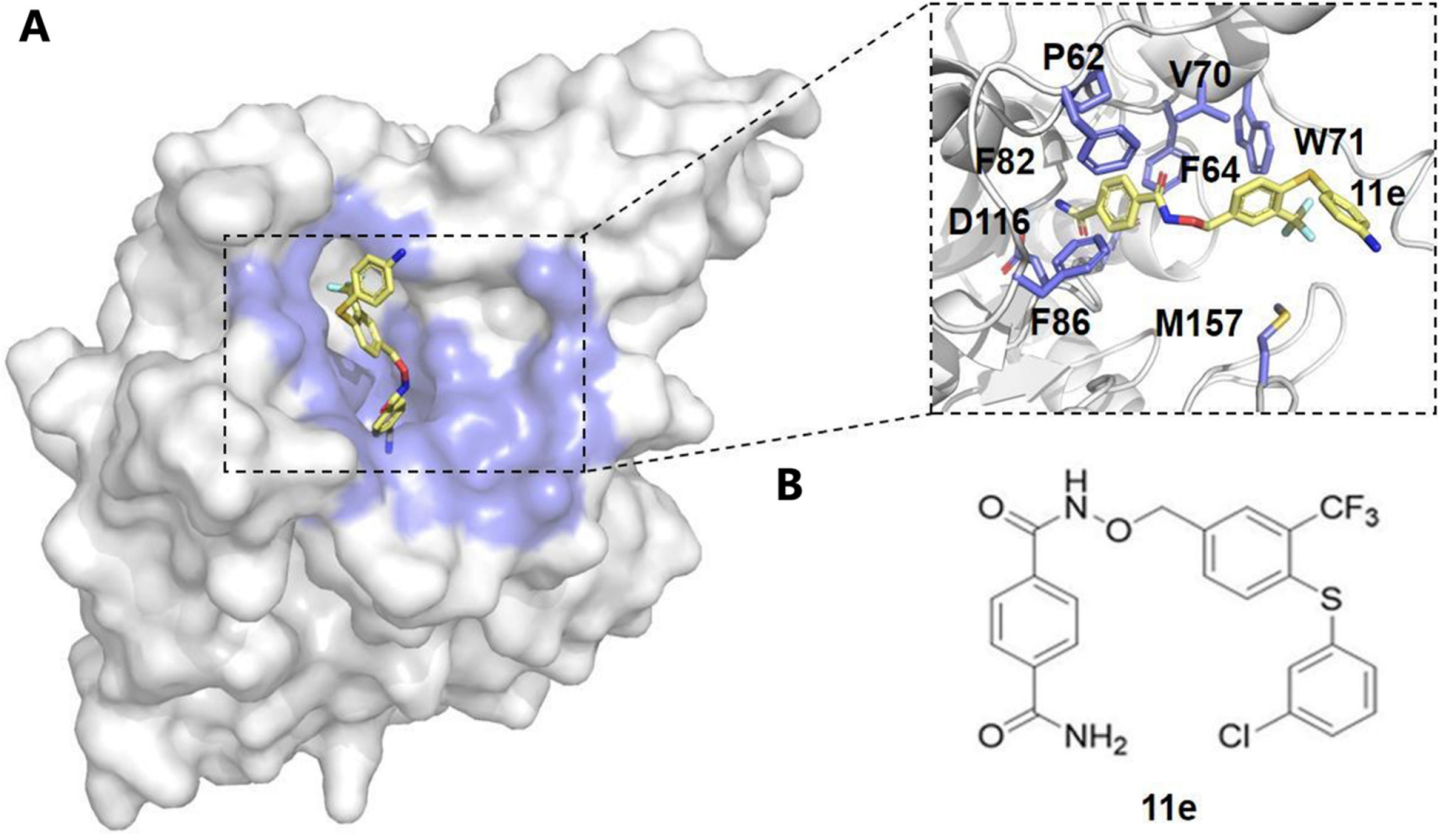
Structural analysis of compound 11e binding to the allosteric site. (A) The surface representation of the protein structure shows compound 11e (yellow) bound to the allosteric pocket, which is highlighted in blue. The inset provides a detailed view of key interacting residues within the allosteric site, including P62, F64, F82, V70, W71, F86, D116, and M157, which are involved in stabilizing the binding of compound 11e. (B) Chemical structure of compound 11e, showing its functional groups that contribute to the interaction with the allosteric site. These interactions suggest a potential mechanism for modulating enzyme activity through allosteric regulation.

Computational methods have been pivotal in identifying allosteric modulators of SIRT6, using techniques such as molecular docking, virtual screening, and MD simulations. Huang *et al.* utilized a combined computational and experimental approach to screen for potential SIRT6 activators.^96^ The Allosite method was used to predict the full enzyme active site of SIRT6, and based on this prediction, over five million compounds were screened.^96^ Through virtual docking, 20 compounds were identified, and their activity was further validated. Among them, MDL-800 and MDL-801 were found to significantly enhance the catalytic efficiency of SIRT6. Shang *et al.* integrated these computational methods to explore the binding of MDL-800 and MDL-801 with SIRT6.^97,98^ MD simulations revealed that MDL-800 notably enhanced the deacetylase activity of SIRT6 and inhibited the proliferation of non-small cell lung cancer (NSCLC) cells by inducing G0/G1 cell cycle arrest.^97^ Furthermore, simulations of MDL-801 indicated that this compound induced conformational changes in SIRT6, stabilizing its active form, particularly through key interactions with residue Met136.^98,99^ Principal component analysis (PCA) and free energy calculations using the AlloSigMA server further highlighted the allosteric coupling between the MDL-801 binding site and the NAD^+^ site, offering detailed insights into its role as an allosteric activator of SIRT6.^98^ Similarly, virtual screening and molecular docking also contributed to the identification of novel inhibitors, such as 11e and compound 8a.^100–102^ Compound 11e was found to disrupt internal signaling pathways and reduce SIRT6 activity, offering a new approach for pancreatic cancer therapy^100^ (Fig. 3). MD simulations revealed that 11e binding induced significant conformational changes in SIRT6, and further free energy and community network analyses demonstrated that 11e binding disrupted internal signaling pathways, reducing SIRT6 deacetylase activity, thus presenting a novel approach for anti-pancreatic cancer therapy.^100^ Compound 8a was identified as a non-competitive inhibitor of SIRT6.^101,102^ Binding energy calculations confirmed the strong interaction between 8a and SIRT6, further supporting its inhibitory mechanism.^102^

Beyond small-molecule screening, advanced computational methods have been used to identify previously unrecognized allosteric sites in SIRT6. Zhang *et al.* employed enhanced sampling MD simulations and Markov State Modeling (MSM) to reveal a cryptic allosteric site, termed “Pocket Z”, within SIRT6.^103^ Through accelerated MD (aMD) simulations, they found that NAD+ binding induces a coupling between Pocket Z and the catalytic domain, altering the function of SIRT6. To validate this novel site, they performed high-throughput virtual screening, leading to the identification of JYQ-42, an allosteric inhibitor.^103^ Binding free energy calculations and per-residue decomposition analysis confirmed the stability of the JYQ-42/SIRT6 complex, validating the druggability of Pocket Z as a therapeutic target.^103^

These studies collectively demonstrate the power of computational techniques—including virtual screening, molecular docking, MD simulations, and free energy calculations—in identifying both activators and inhibitors of SIRT6. Through these methods, researchers can gain deeper insights into the allosteric regulation of SIRT6 and facilitate the rational design of novel modulators for cancer therapy.

### 3.2 Computational approaches in targeting MEK allosteric sites for cancer therapy

**Fig. 4:**
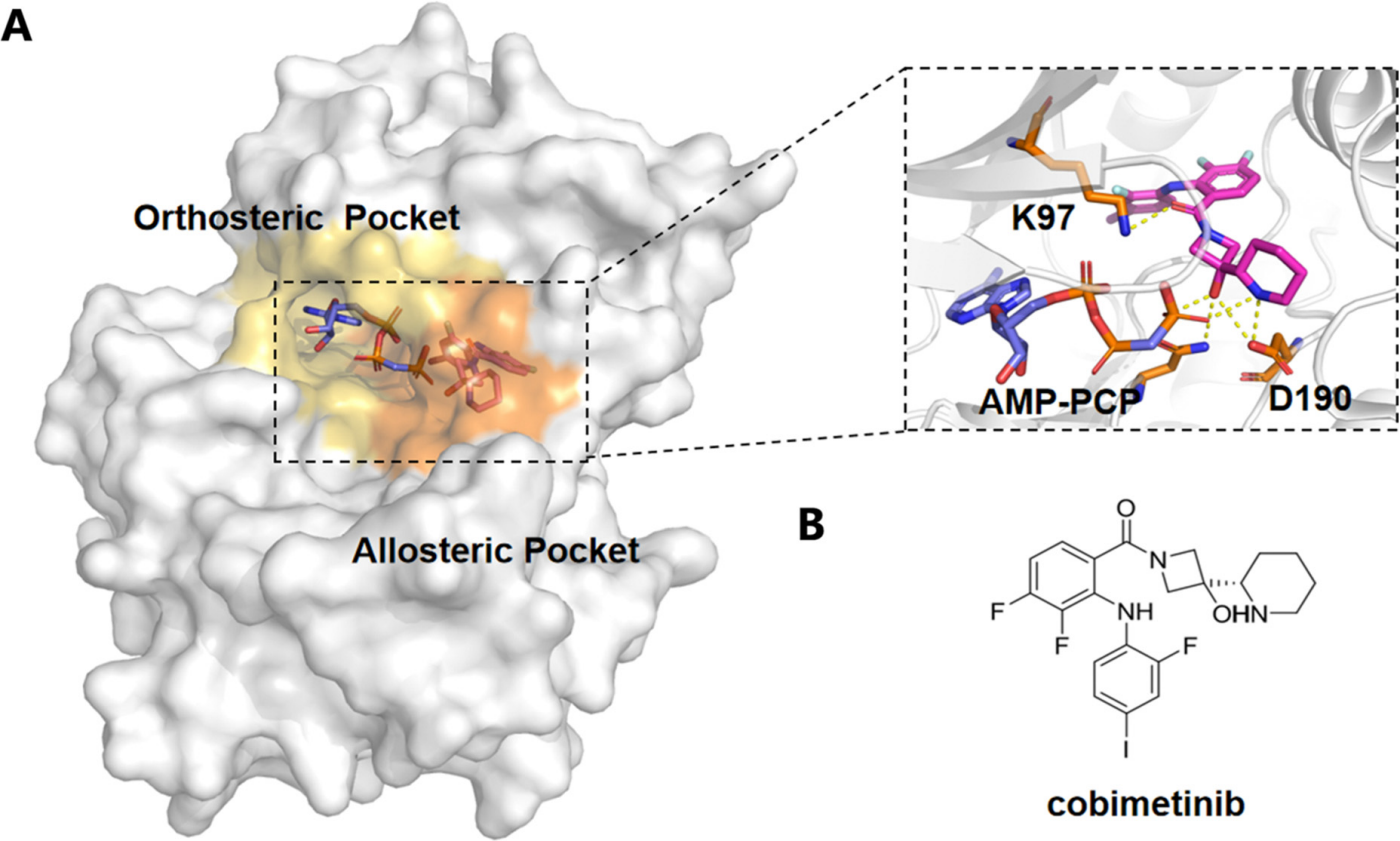
Structural analysis of cobimetinib binding to the orthosteric and allosteric pockets of the enzyme. (A) Surface representation of the enzyme showing the orthosteric pocket (yellow) and allosteric pocket (orange) with cobimetinib binding across both sites. The inset highlights the detailed interactions of AMP-PCP (blue) and cobimetinib (magenta) within the binding region, showing critical residues such as K97 and D190 involved in ligand coordination. (B) Chemical structure of cobimetinib, illustrating its pharmacophore elements that interact with the enzyme's active and allosteric sites.

MAPK/ERK kinase (MEK) is a critical dual-specificity kinase in the Raf-MEK-ERK signaling cascade, playing a pivotal role in the regulation of cell proliferation, survival, and differentiation.^92,104^ MEK1/2 are the only known substrates of Raf kinase, involved in the activation of downstream ERK1/2.^104,105^ Overactivation of MEK1 and MEK2 is closely associated with various inflammatory conditions and approximately 30% of human cancers, making them key targets for anticancer drug development.^92,106^ To date, four MEK allosteric inhibitors—Trametinib, Binimetinib, Selumetinib, and Cobimetinib—have received FDA approval for effectively inhibiting MEK1/2 activity^92,107–109^ (Fig. 4).

A recent study by Mudedla *et al.* employed molecular dynamics (MD) simulations to investigate the impact of several MEK1 allosteric inhibitors (such as selumetinib, trametinib, cobimetinib, and CH5126766) on MEK1.^110^ The results revealed that these inhibitors bind to an allosteric pocket near the MEK1 αC helix, restricting the flexibility of the MEK1 activation loop, particularly preventing Ser222 from approaching ATP, thereby stabilizing its inactive conformation and blocking Raf-mediated MEK activation.^92,110^ This mechanism leads to the suppression of MAPK pathway signaling. The free energy perturbation (FEP) method, which combines free energy calculations, accurately predicted the binding affinities of these inhibitors and showed strong correlation with experimental IC50 values. By employing MD simulations, the study provided deeper insights into how allosteric inhibitors block MEK1 activation, offering valuable guidance for the design of more selective and potent MEK1 inhibitors for cancer therapy. Furthermore, Di Fruscia *et al.* utilized fragment-based and virtual screening strategies to target the allosteric site of MEK1.^111^ They constructed a fragment library and identified 142 potential binders through 1D NMR screening.^111^ They constructed a fragment library and identified 142 potential binders through 1D NMR screening.^111^ These findings demonstrate the effectiveness of fragment-based screening in identifying novel allosteric modulators.^111^

In summary, MEK plays a pivotal regulatory role in the Raf-MEK-ERK signaling pathway, and its allosteric site has emerged as a critical target for anticancer drug development.^112^ Through computational approaches, including virtual screening, fragment-based screening, and molecular dynamics simulations, researchers have successfully identified and optimized MEK allosteric modulators, while gaining in-depth insights into the molecular mechanisms of MEK1 and its interactions with inhibitors.^92,104,111,112^ These findings provide essential guidance for the design of more efficient and selective drugs, paving the way for new strategies in cancer treatment.

## 4. Conclusions: advancing computational methods for allosteric drug discovery

Allosteric activators in enzymes represent a promising avenue for drug discovery, offering distinct advantages over traditional orthosteric drugs, such as increased specificity and reduced off-target effects. This review highlights the integration of computational approaches, including MD simulations, enhanced sampling techniques, NMA, evolutionary conservation, and ML, to identify and characterize allosteric sites in enzymes. These approaches collectively provide a comprehensive framework for understanding enzyme dynamics and pinpointing potential regulatory sites for therapeutic intervention.

The application of advanced computational tools like PASSer,^46,80,81^ AlloReverse,^34^ and AlphaFold,^32,84^ in combination with molecular docking and free energy calculations, has successfully revealed cryptic allosteric sites and provided a deeper understanding of their regulatory mechanisms. Furthermore, the identification of allosteric modulators, as demonstrated in the studies of SIRT6 and MEK, underscores the therapeutic potential of targeting allosteric pathways, particularly in oncology.

The advances in computational techniques not only enhance our ability to predict allosteric sites but also provide critical insights into the molecular mechanisms of allosteric regulation. This progress paves the way for rational drug design targeting allosteric sites, with the potential to develop more selective and efficacious therapeutic agents. Future research should optimize these computational methods, integrate experimental validation, and explore the broad applicability of allosteric modulators in diverse therapeutic areas, ultimately bridging the gap between computational predictions and clinical outcomes.

## Author contributions

R. Zhu: conceptualization, writing – original draft, review & editing. C. Wu: writing – original draft, review & editing. J. Zha: writing – original draft, review & editing. J. Zhang: conceptualization, funding acquisition, supervision, writing – review & editing. All authors have approved of the final draft of the manuscript.

## Conflicts of interest

There are no conflicts of interest to declare.

## Data Availability

No primary research results, software, or code have been included and no new data were generated or analyzed as part of this review.
